# Optimization and planning of operating theatre activities: an original definition of pathways and process modeling

**DOI:** 10.1186/s12911-015-0161-7

**Published:** 2015-05-17

**Authors:** Simone Barbagallo, Luca Corradi, Jean de Ville de Goyet, Marina Iannucci, Ivan Porro, Nicola Rosso, Elena Tanfani, Angela Testi

**Affiliations:** Bambino Gesù Children’s Hospital, Piazza San Onofrio 4, Rome, 00165 Italy; Nextage s.r.l, Genoa, Italy; Department of Economics and Business Studies, University of Genoa, Genoa, Italy

**Keywords:** Clinical pathways, Business Process Modeling, Operating room planning and scheduling

## Abstract

**Background:**

The Operating Room (OR) is a key resource of all major hospitals, but it also accounts for up 40 % of resource costs. Improving cost effectiveness, while maintaining a quality of care, is a universal objective. These goals imply an optimization of planning and a scheduling of the activities involved. This is highly challenging due to the inherent variable and unpredictable nature of surgery.

**Methods:**

A Business Process Modeling Notation (BPMN 2.0) was used for the representation of the “OR Process” (being defined as the sequence of all of the elementary steps between “patient ready for surgery” to “patient operated upon”) as a general pathway (“path”). The path was then both further standardized as much as possible and, at the same time, keeping all of the key-elements that would allow one to address or define the other steps of planning, and the inherent and wide variability in terms of patient specificity. The path was used to schedule OR activity, room-by-room, and day-by-day, feeding the process from a “waiting list database” and using a mathematical optimization model with the objective of ending up in an optimized planning.

**Results:**

The OR process was defined with special attention paid to flows, timing and resource involvement. Standardization involved a dynamics operation and defined an expected operating time for each operation. The optimization model has been implemented and tested on real clinical data. The comparison of the results reported with the real data, shows that by using the optimization model, allows for the scheduling of about 30 % more patients than in actual practice, as well as to better exploit the OR efficiency, increasing the average operating room utilization rate up to 20 %.

**Conclusions:**

The optimization of OR activity planning is essential in order to manage the hospital’s waiting list. Optimal planning is facilitated by defining the operation as a standard pathway where all variables are taken into account. By allowing a precise scheduling, it feeds the process of planning and, further up-stream, the management of a waiting list in an interactive and bi-directional dynamic process.

## Background

Providing a good quality of care is a primary objective -in fact a priority and a duty- of healthcare institutions worldwide. The rapid development of medical technologies and their application to a continuously wider spectrum of diseases, conditions, and patients; the unstoppable growth of the health related requests of a population that is also growing in size; poverty and ageing; has all economically challenged health systems and insurance priorities around the world, to a point where their sustainability is nowadays very much questioned in every country, and where most costs have been covered by the government. In these countries, a direct contribution is now increasingly being asked to anyone benefiting from a care and complementary cover, and whereby private insurance is being incentivized. Even in countries like the USA, where private companies cover 2/3 of health spending, the discussions of increased state aid in favor of a large part of the population has created major social and political tension. National health spending around the world varies between countries, but it is constantly representing huge amounts of money per country between 5 % and 17 % (USA) of the GDP in northern hemisphere countries (17 % in the USA, and around 10 % in most large European countries) [1]. It is estimated that hospital budgets account for almost half of all spending in most health systems. Within the hospitals, the most important reason for hospital admission nowadays, are procedures or surgical interventions, and the operating room areas are considered to be the most expensive facility, consuming a large part (>40 %) of their annual budget [2].

In the last two decades, in a general and economical context of delivering a better quality of care with limited resources, and ultimately, at the lowest cost, while facing with the growing demand for procedure and interventions, one hospital unit has emerged as of being of particular interest for management teams and has been targeted for reaching the inferred objective: that being, the operating room area. Many healthcare institutions have thus been researching tools for optimizing patient flow, studying the various processes in the operation area, and the usage rate of the operating theatre. All of this, while containing, or reducing where possible, the costs. This is clearly a call for increasing efficiency and for the development of advanced methods for planning and scheduling of hospital procedures and Operating Room (OR) resources. The goal of optimization has indeed become a central theme in modern hospital management [3–5]. This blueprint for OR efficiency started as early as the eighties, when Magerlein and Martin [6] had already analyzed the basic concepts. The following 30-year period of time has been paved with a large amount of research studies, in an attempt to address the irresoluble problem of the best management of operating theaters, while additionally organizing the planning of surgery room activities. From a given definition of the problem, and also from a management and procedural point of view, various approaches have been attempted; excellent recent reviews are now available by Cardoen et al. [7], Guerrerio and Guido [8] and Spyropoulos [9].

The planning and scheduling of the processes of an operating room area are known to be a very complex task. This is because of the many constraints (sometimes opposite in their objectives, but as a way of increasing quality and satisfaction, but with the lowering of costs, whilst limiting resources). The inherent variability of the case mix (specialties, interventions), and also even of each precise type (the particularities and the characteristics of each patient), and the fact that many different actors are involved with sometimes conflicting interests are all trying to achieve different objectives.

From the analysis of the literature it appears that OR planning and scheduling studies have been oriented mostly towards the management of elective patients, with very little research for non-elective situations. This is when, in fact, an important part of the so-called “elective” work consists of non-emergency patients (i.e. set forth and to do on the same day) that need an operation within a day to a week. This is a reality and is a major last minute disruption in many hospitals, which is rarely taken into account.

As pointed out by Cardoen et al. [7], many studies have also considered only single-objective case mix models, not allowing to adequately represent such a complex problem as the planning and scheduling in the real world is. Although Cardoen et al. [7] stimulate for studying the stochastic aspect of activity durations and their impact on the operating room flow, they also question the increased computational complexity that would be necessary to achieve it. Guerriero and Guido [8] and Spyropoulos [9] highlighted another research paradigm; many, indeed too many studies, have been proposed mathematical models excessively, or only in the majority of cases, applied to “the process” of surgical planning and scheduling, which often did not provide practical clues for solutions to be adhered to in operating theatres.

Lastly, most past studies that have been dedicated to operating rooms planning and scheduling have been giving excessive attention on immediate problems that are related to the flowing of processes in operating areas on the day of surgery, rather than on the medium-term (a month in advance) and close-term (a week in advance), when considered from a planning perspective. By looking excessively and closely, part of the problem may escape an understanding. Reframing the approach, or looking from “out of the frame”, might be part of the solution. This might even allow for other developments, as predicting the work is a major information problem for the healthcare system; i.e., being able to calculate for the medium term and the future planning of resources (the use of material and facilities, the employment of operative teams, and other auxiliary workers).

In this general context, the present work consisted first in defining a standardized “pathway” representing the operating process, defined as the sequence of all elementary steps between “patient ready for surgery” to “patient operated”, and its specific duration. The pathway has been expanded to the point of including the different specific surgical timings and human resources use, mirroring what is really used in hospitals.

The adoption of standard pathways for the care processes (i.e., clinical pathways), brings significant benefits, including: improved outcomes for the patient, an improvement of interdisciplinary work, a better management of clinical resources, better hospital efficiency, and a continuous improvement in the quality of care [10, 11]. As well defined by Vanhaecht et al. [10]: “The main method to (re) organize a care process is the development and implementation of care pathways”.

To stimulate a switch from research to practical implementation, the “Business Process Modeling Notation” (BPMN 2.0) has been adopted as reference standard to describe the operating pathways. Indeed, one of originality and uniqueness of this study is using the information gathered from the BPMN analytical tool, for developing a mathematical optimization model aimed at planning the OR activities and determining, for each day and each OR, the set of patients scheduled for surgery.

This paper is thus intended to show the benefits of an integrated BPM and optimization approach, as a valuable support for the decision-making processes involved in the management of operating rooms. The definition of the operating pathways and process modeling, allows for a better description of the decision-making requirements that form the basis for a subsequent optimization modeling phase.

The paper has been organized as follows. In the next subsection a detailed description of the problem under study is firstly given. Section 2 describes the three step methodology used in Bambino Gesù Children’s Hospital (Rome, Italy) to define the standardized surgical pathways, the resource use time and to tasks allocation, as well as the mathematical model for the OR planning optimization. The results of the application of the model to real clinical data and waiting lists are given in Section 3 and compared retrospectively to the current practice. In Section 4 the discussion of the research results is provided, while conclusions and future studies directions are reported in Section 5.

### Problem description

To understand the problems of OR planning and scheduling performing, it is necessary to present in its wholeness the “operating process”, i.e. the whole “pathway” of a patient undergoing intervention. Before any hospitalization, a patient must be seen by the surgeon. He or she decides what type of surgery is indicated, the window of time for doing it (priority), and possibly, a predicted duration time. First of all, the patient will be registered on a waiting list, and later, will be attributed to a calendar date. Surgeons usually know in advance on what day and in which room they can operate according to a planning as defined by the hospital manager – i.e., a general operating schedule, often called a “Master Surgical Schedule” (MSS), specifying the distribution of operating room resources between surgeons. Some rooms may have technical and logistic specifications that may be relevant for some intervention types. The interval between the first and second step may vary in accordance with the relative priority of the patient’s procedure, when compared with that of other patients registered on the waiting list. That having being said, the waiting time of the latter patients will have to be taken into account.

A set of information about the patient and/or the procedure may have already been collected at this stage, although this may be done at a further stage (the specific instruments needed, the particular problems of the patient, complementary contemporaneous procedures, the predicted duration of the operation, etc.). Usually, and at some point during the interval, an anesthetist will confirm the operability of the patient, the need for complementary checks on the day of admission, and the possible need for an intensive care stay after the procedure. Preferably, once a date for the operation has been proposed, there will be no changes as a matter of patient satisfaction – unless there is no alternative for dealing with such priorities. A more precise timing of the operation, i.e., the possible hour of the day for admission into the operating theatre (i.e. the “scheduling”), is done variably within a week, or a day before, the day of the procedure.

Hospitalized, generally the day before the intervention, the patient will be checked for a completeness of the information in view of the operation, and for his or her fitness for undergoing the procedure (a visit from both the surgeon and the anesthetist). If any contraindication for the operation may be found, the patient would be canceled from the schedule at that point. At this time, a re-organization of the schedule and the calling in of another patient in would be attempted. If the patient needed an admission into intensive care after the operation, the bed will be booked in advance, and the availability will be checked again the next morning, before transferring the patient to the operating theater. If a bed is not available, the patient may be left on stand-by on the morning of the day of the operation, until a bed is guaranteed. During the interval, and the delay, the schedule may be revisited and other patients operated on while the aforesaid patient is waiting. The patient may be postponed to another day eventually, with the related schedule of another day reorganized in turn. For the intervention itself, various human resources implied in the process (surgeons, anesthesiologists, nurses, carriers, etc.) are scheduled and organized in order to synchronize the procedure. Other procedures being possibly added, implies that other teams and logistics are to be called in to contribute at specific times (the technician of radiology for radiography, the anatomopathologist for diagnostic biopsies, a second surgical team for complementary interventions). The time necessary for preparing the room and the patient varies in accordance with the material to prepare, and also with the need, or not, for the anesthetist to perform a specific preparation for the patient (central venous catheter, epidural anesthesia, etc.). The use of a preparation room before admission into the operating theatre room, the use of a postoperative recovery room, or the immediate transfer into intensive care, all have a direct effect on the occupation of the operating room. Its availability for another procedure, and thus, directly affects the flow of patients through the operating area. Other bottlenecks in the process are well known by managers: the availability of a carrier for patient transfer, the time for the transfer, the waiting time for any specialist involved, can all be delayed by other procedures, with an operation duration going much under, or over, the predicted time.

In the real world, patients are coming for an intervention as a continuous flow, each with his, or her own, characteristics and relative priority. This creates a permanent need for revising the planning in the medium term, and in the schedule, until even a day before the operations.

Attempts at planning on a “first come first served” basis (open-booking) in the past have been incapable of adequately addressing the need of all of the surgeons in the hospital, and more importantly, addressing the problem of priority and relative urgency of patients. Most hospitals use now a MSS with a “block-booking” type of functioning. This is where surgeons are allocated, each week, a given space/time that is authorized on the basis of their mean needs. Although this raises other issues about how managers can establish an equitable MSS, this is the most common way of doing so worldwide, with the advantage of allowing the balancing of priorities for each, and all, surgical specialties [12].

However, even within the “blocks” allocated to a surgeon, the way he distributes his patients is continuously revised when new patients enter the waiting list. This meant that most surgeon’s would delay the planning and the scheduling to the latest possible date before the operation, unless they could function with any ample space as a leftover (so they could easily introduce one more patient, any time close to a given date). In turn, with this type of functioning, in other words, the decision-making process, is very much left to be guided by unspecified, intuitive criteria, that may vary between doctors [13]. In this context, the use of the corresponding operation room time, is usually under-optimal, since often, not all usable time is used [14, 15].

Lastly, come whatever, and last minute cancellations for a wide range of reasons add more disruption to the OR planning and the need to rescheduling some surgical cases to other days [16, 17]. This is not to speak about the real emergencies of the day, which may happen to disturb an elective operating area, even when the hospital has defined a specific operating room for such cases.

From this reality, stemmed this project. Specifically, the organizational difficulty and the complexity, depend on both the high number of constraints, and the variables to consider, and in terms of manufacturing the variability of the “product” (the patient and the care to provide) and the inherent uncertainty of the duration of the procedure, and of the resources that will be used, all of that makes the fact of the “manufacturing” (the planning and the scheduling of care delivery) extremely hazardous [2, 18, 19].

This observation triggered us to consider a different approach, where the heterogeneity of the interventions and the processes is reduced to a standardized single “pathway”, with the path integrating most of the variables that could affect patient flow and the process itself in the OR [20]. For doing that, a revitalizing “out of the frame” vision was necessary, leading one to consider the patient’s pathway on a larger scale, from the insertion onto the waiting list, rather than at a later step, along with the scheduling, as many previous studies have done.

By doing that, this research has also approached two other major difficulties of real-life and of previous studies. How to approach and to take into account the inherent uncertainty of surgery duration even using a deterministic optimization model, and how to possibly achieve an automatic planning and scheduling for the creation of OR schedules and waiting list management in an interactive and bi-directional dynamic process.

## Methods

The study was carried out by a multidisciplinary team working at - or with - the Bambino Gesù Children’s Hospital located in Rome (Italy). The hospital is both a national tertiary referral center and a local and regional hospital, and is a center of excellence for the research and the effectuation and innovation of pediatric care. Interestingly, although the care must be delivered to a wide range, in terms of the complexity of cases, it is organized in relatively small OR areas, that facilitate the analysis, the modeling, and possibly, also the implementation of a solution for the future.

**Fig. 1:**
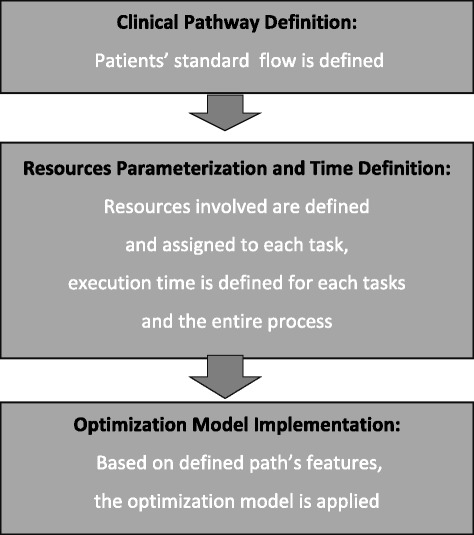
Process evaluation scheme: a 3 step methodology

In a few words, the research process was run in three major sequential steps, complementary of each other, and allowing for: a) an in-depth and complete definition of the pathway first, b) a detailed parameterization of all resources, tasks, and times, related to the processes, and c) an optimization model for developing OR schedules (Fig. 1).

As a first step, the clinical pathway was defined as a standardized pathway representing the operating process (defined as the sequence of all of the elementary steps between the “patient ready for surgery” to the “patient operated upon”) and its specific duration. This has been expanded to the point of including all of the different specific surgical timings, and the use of human resources, mirroring what is really utilized in a hospital. The studied OR area was a dedicated small complex one with only three operating rooms, devoted to the elective surgeries of the General Surgical Department under study. The limited size of this structure and of the related activity was considered to be a contributing factor for facilitating the analysis and the modeling.

The three steps of the approach are below described in details.

### Defining the standard pathway

Standardization can be defined as a process of re-engineering, which aims to obtain the same behavior of a given process in all instances. Ideally, if all processes had the same behavior, it would also be very easy to apply an integrated management system for them. However, if a process contains a lot of exceptions, then it will be much more complicated to support this definition and its management. This is the case of standardization at the hospital level. Given the particularity of our case, the definition of a precise and comprehensive process acquires even more importance; such a definition is fundamental for both process standardization and its subsequent management.

Standardization can be defined as a process of re-engineering, which aims to obtain the same behavior of a given process in all instances. Ideally, if all processes had the same behavior, it would also be very easy to apply an integrated management system for them. However, if a process contains a lot of exceptions, then it will be much more complicated to support this definition and its management. This is the case of standardization at the hospital level. Given the particularity of our case, the definition of a precise and comprehensive process acquires even more importance; such a definition is fundamental for both process standardization, its subsequent management and the development and deployment of needed IT tools.

Our purpose was to redefine the overall surgical processes based on already available information and know issues about existing procedures. Being a top-down modelling approach not requiring to model low level details, state-of-art process mining techniques to automatically infer processes definition from execution samples were not required [21]. On the other way, the process modelling took a less relevant role in our project, being the operating theatre modelling and optimisation work the prominent one.

Before choosing a modelling technique, a review of available solutions has been done. Our target was to identify a technique being able to define a standardised communication framework between staff with a computer science background (IT and software developers), clinical staff (surgeons, anaesthetists, nurses, management) and management. Most important selection criteria was (i) the ability to model processes and events with a good formalization level (in the perspective of a future software development activity), (ii) the ease of use by non-IT people (least possible complexity of terms and vocabulary), (iii) its diffusion and maturity in the clinical context (to allow for ease of communication in the community and future use among time).

Example of software-oriented modelling languages are EXPRESS (ISO 10303–11), IDEF, UML, BPMN. EXPRESS [22] is a standard for data interoperability in long-term large projects, especially in the aerospace and military industry. It is focused on data description and data operability instead of process description. IDEF is a set of techniques used to model processes (the most important ones are IDEF0 and IDEF3 [23]). It was the approach of choice in the 1990s. It is a robust industry standard however it has been developed long before the diffusion of modern computer-aided software engineering (CASE) tools and it is less flexible in terms of business process management. Also, the graphical representation is more complex than in most recent tools. Among them, BPMN and UML AD (Unified Modelling Language Activity Diagrams) are the most common. Both are well understandable by non-technical people and designed to model business processes. However, while BPMN is slightly easier than UML AD (some components are modelled using only one symbol in BPMN and using a group of symbols in UML AD) and provides a direct mapping to business process execution languages (BPML) while UML needs intermediation [24].

This is what led us to define the pathway solution by using the standard BPMN (2.0 Version). BPMN is a language specifically designed to model business processes and their management [25]. There are many references concerning BPMN’s adoption in healthcare for various applications: medical assistance; specific hospital processes; structure application, and technique utilization [26–28]. The subsequent definition of a new and innovative standard 2.0 [29] in 2011 has guided our choice. Other important features of the BPMN are its clarity and comprehensibility by all kinds of specialists: such as computer scientists, IT staff, healthcare workers, and management personnel. This was the main reason that led to its adoption by the team, as the standard for process definition, instead of other solutions such as, for example, the Petri Nets [30]. The main characteristic of BPMN 2.0, that led us to adopt it, is that this modeling technique simplifies and facilitates future software implementation, which will be needed to manage and optimize the process. BPMN is essentially a derivative of the formalism of a flow chart, but with some additions and modifications, which overcome certain limitations in modeling business processes, and enable process adaptation, process flexibility, and process evolution [31]. It allows one to construct process diagrams (BPD - Business Process Diagrams) representing graphs, or networks made of “objects” exhibited by the process activities, connected by control flows, which define the logical relationship, the dependencies, and the order of execution of the activities. The use of the BPMN standard can also define a specific workflow, for the process under investigation, and its subsequent development including computerization, with resource management, and the definition of the actors involved.

However, operating areas are highly complex environments in terms of planning, scheduling, cost effectiveness, and optimization of processes. Not surprisingly, although the BPM approach has been recognized as a standard for process modeling in healthcare organizations [32–34], its use as a unique tool for finalizing studies up to a practical and real step, has been limited when addressing a complex OR environment.

**Fig. 2:**
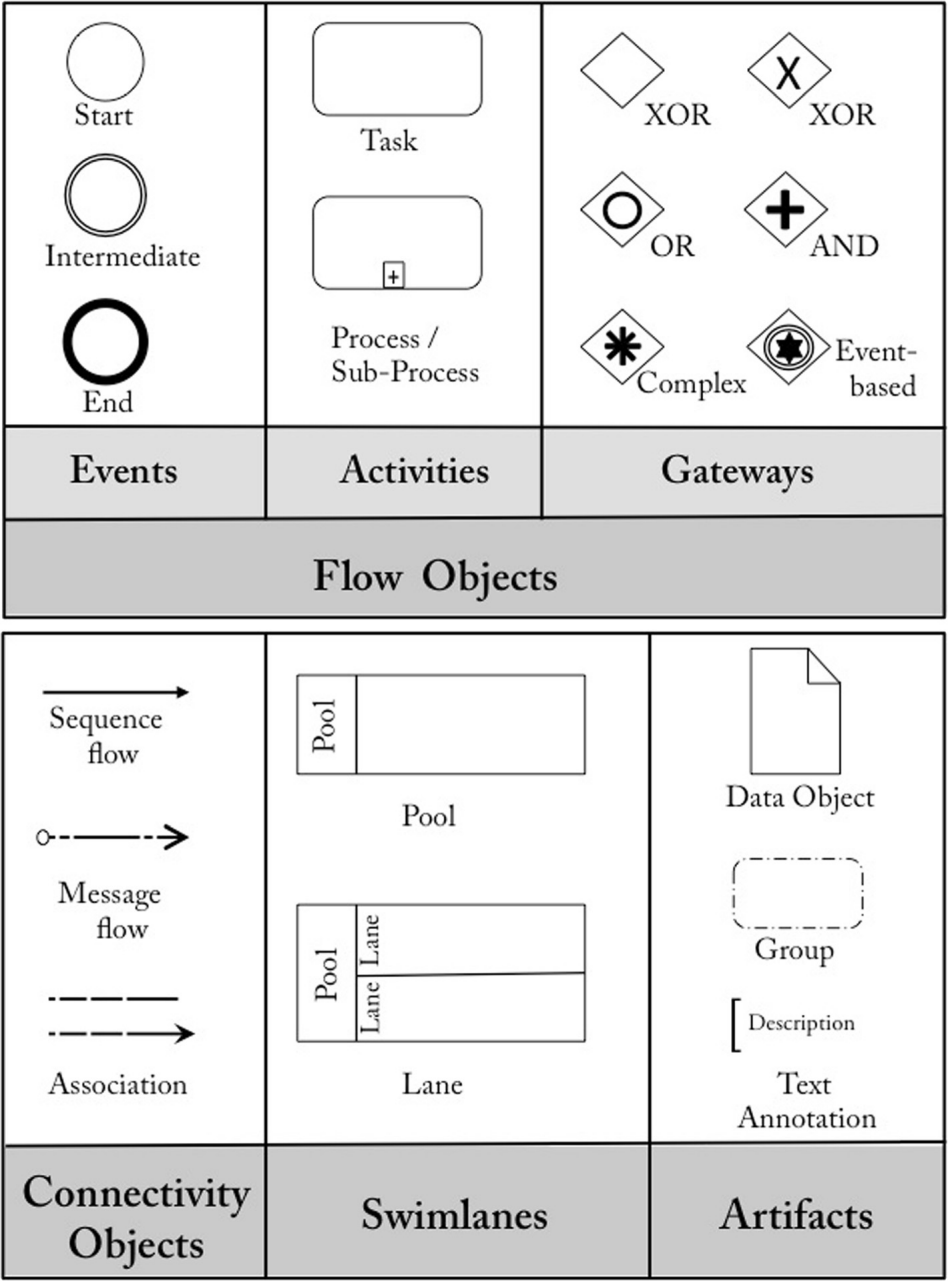
Core elements in BPMN

The core of BPMN’s standard is categorized into four groups by various elements [35], as shown in Fig. 2.

**Fig. 3:**
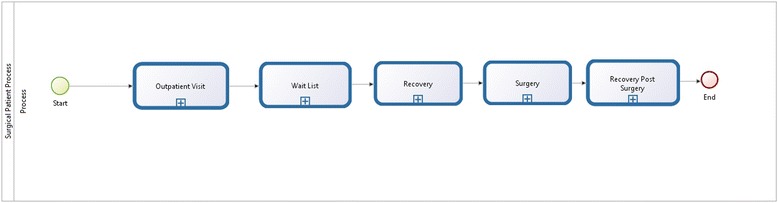
General process followed by a patient in the hospital

The work began with the writing of a much detailed definition of a standard operative path, and in particular, the path followed by a patient from the ward, ready for surgery, and then back to the ward when the surgery ends. This path is just a part of the general process of a patient in a hospital as shown in Fig. 3.

**Fig. 4:**
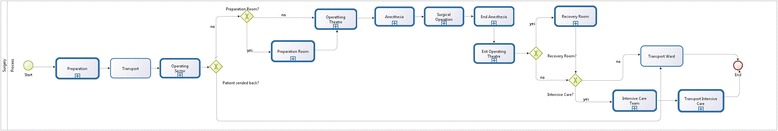
Specific surgical process

In particular, the general process starts at the outpatient visit (a consultant examines the patient and suggests surgery), goes through the waiting list and admission periods, and ends with surgery and a post-surgery stay in the hospital. After an analysis of the generic processes, we focused on a particular definition of the surgical process, for the reasons as previously described. With the assistance of the hospital’s physicians (the surgeons and the anesthetists), the team defined a detailed surgical flow as shown in Fig. 4.

The surgical flow chart is divided into twelve main tasks that represent the process activities. They are coded from 1 to 12 and each number represents a single task: Preparation, Transport, Operating Sector, Preparation Room, Operating Theatre, Anesthesia, Surgical Operation, End of Anesthesia, Exiting the Operating Theatre, the Recovery Room, Transport to Intensive Care, and Transport to the Ward.

The general flow-chart, which is valid for all the patients needing any type of surgical operation, starts with their preparation in the ward. Afterwards, the patients move to the operative area, where there is a possibility that they may spend some time in the preparation room, before being moved into the operating theatre. The next steps describe the activities during anesthesia, surgical, and post-surgical operations. The end of the process is represented by the exit from the operating theatre, which can occur in three different ways: transport to the recovery room, transport to intensive care, or directly to the ward. In a first analysis, it is possible to define several peculiarities of both the whole operative path and some specific blocks. First of all, the main characteristics of the path are linearity and consequentiality up to the exit from the operating theatre. Second, as we have previously stated, the patient can follow one of three different ways, depending on his or her condition. Transport to the intensive care unit takes a specific route, because it is used when a patient is in a critical situation. As we will describe later on, this specific path influences both time and resources. Another particularity of the process concerns the so-called ‘exit’ points of the surgical flow, which are used if something “goes wrong”. At such points, the patient should be moved straight to the ward, and the scheduling program and the wait list insertion reactivated. The possibility of the “Preparation Room” being used, or not, must also be taken into account and integrated as a possible step within the pathway, either before moving to the operating theater, or on the way back to the ward. This room can be used to prepare the patient for the surgical intervention, but its utilization depends upon the patient’s condition (age, allergies, risks, etc.) and on the specific anesthetic requirements. Whether this room is used, or not, clearly affects the operating theatre’s utilization time. For this reason, it is important to know in advance, whether it will be used, in order to precisely define an occupation time for each operating theatre.

Note that, the patient flow is defined with a view to future integration with a complete OR planning step. A specific flow and timing for the operating theatre cleaning is also defined, since it is another crucial factor for the definition of an operating theatre’s occupation time. The theatre cleaning duration varies according to the different types of anesthesia chosen by the team. The general workflow definition has also been integrated with specific information, identification, resources, and the timing for each task, in order to obtain a complete solution for the standard process and flow (see Section 2.2).

### Parameterization of resource use and time allocation to tasks

After defining the whole process and determining each single task, we focused our interest on assessing the human resources involved, all along the defined pathway, and at each step. Table 1 represents a list of each human resource involved, while Table 2 specifies in which task they are involved. Table 2 also defines the principal actor of the task, which should be the person in charge of the specific pathway.

**Table 1:** Resources involved in the process

Resource	Code
Ward Nurse	A
Ward Doctor	B
Surgeon	C
Anesthesiologist	D
Operative Sector Nurse	E
Operating Theatre Nurse	F
Technician	G
Stretcher-Bearer	H
Preparation Room Nurse	I
Intensive Care Team	J
Operating Theatre Cleaner	K

**Table 2:** Resources involved in each task and principal actor

Tasks	Resources	Principal actor
Preparation	A, B, C ,D	B
Transport	A, H	H
Operating Sector	H, D ,E	D
Preparation Room	D ,I, G	D
Operating Theatre	C, D, F(3)	C, D, F
Anesthesia	C, D(1/2), F(3)	D
Surgical Operation	C(2), D(1/2), F(3)	C
End Anesthesia	C, D, F(3)	D
Exit Operating Theatre	D, E, F(2), K	D
Recovery Room	D, F, I, J, K	D
Transport Intensive Care	J, D, F	J
Transport Ward	E, H, A, B	H

This allows one to define exactly ‘who’ is involved and ‘where’ in each part of the process is he or she active, and also, who is the principal actor. For a perfect management of the process, it will be necessary to assure of its integration with the hospital work shifts. Furthermore, by studying the operative practices, and with the hospital doctors’ fundamental support, we have defined the information necessary to go ahead in the process and in each task (Table 3).

**Table 3:** Information needed for the process

	Info
1	Particular Anesthesia
2	Preparation Room Used
3	Particular Room or Instrumentation
4	Particular Patient
5	Allergies or Particular Risk
6	Particular Patient Position
7	Technician
8	Multiple Operation
9	Recovery Room
10	Intensive Care
11	Type of Anesthesia
12	EOT (Expected Operating Time)

As previously was done for resources, it has also established when it is necessary to have this information (Table 4).

**Table 4:** Information needed for each task

Tasks	Info
Preparation	No Info
Transport	No Info
Operating Sector	2, 4, 5
Preparation Room	1, 2, 4, 5, 11
Operating Theatre	1, 4, 5, 11
Anesthesia	1, 2, 3, 4, 5, 6, 11
Surgical Operation	3, 6, 7, 8, 11, 12
End Anesthesia	4, 5, 6, 9, 10, 11, 12
Exit Operating Theatre	4, 5, 10
Recovery Room	9, 10
Transport Intensive Care	10
Transport Ward	No Info

The uncertainty of the duration of an operation is a major problem for scheduling and this has a relevant impact in forecasting and optimizing operating theatre occupation in general. Because each component of the “path” is concerned, we focused our interest on the analysis of the specific sub-timings of the operating sub-processes and not only on the process in general. In the first instance, we described a standard process, usable for every generic patient requiring surgery. We then focused on the diversification of this flow for the various types of intervention, limiting the number of variations, in order for us to define a customized process that would fit each patient flow, and was based upon each particular intervention, the human resources required, and the specific timing of events. To do that, we parameterized certain variables in order to codify the different choices made by the clinical staff. Each significant choice was allotted either a precise execution time, or a value in % representing its incidence on the time of the related process. As a result, the modeling intrinsically includes variations of procedure times and is able to establish a specific duration for any particular process, and also in turn, for the occupation of the operating theatre. One of the most important timing parameter definitions has been that that is associated with the type of anesthesia (Table 5).

**Table 5:** Definition of different types of anesthesia

Code	Features	Preparation room use
A	No Intubation	NO
B	General Anesthesia and Intubation	NO
C	B + One Procedure	NO / YES
D	B + More Than One Procedure	NO / YES

(For A and B types the preparation room is not used; for C and D, it depends on the surgeon’s decision)

For this purpose, “anesthesia” was divided into four types “A, B, C, and D”, on the basis of their specific characteristics (i.e., with or without intubation) and on the basis that certain procedures (i.e., the central vein or the epidural catheter positioning) were associated. All of the parameters were directly linked with the intervention times. As a result, each anesthesia type was in the end, not only descriptive, but also, specifically associated to a specific time of execution and a different use of human resources. Finally, each pathway, for any type of operation, can be associated with a defined and specific type. We believe that this classification is simple and represents the most anesthetic pathway in current practices worldwide.

The parameterization of procedures allows for a fine, but not too an extensive modeling of possible cases, together with their impact on operating room occupancy, and upon various staff requirements. The choice affects various factors, and in particular: anesthesia induction duration, the preparation of the room use, room cleaning duration, and human resource utilization, as is shown in Table 6.

**Table 6:** Number of anesthesiologists involved during induction, operating and awakening task and induction time, patient preparation and cleaning time for each type of anesthesia

Code	Induction (Head Count)	Operating (Head Count)	Recovery (Head Count)	Induction time (in minutes)	Patient preparation and cleaning time (in minutes)
A	1	1	1	10	15
B	1	1	1	15	20
C	2	1	1	30	40
D	2	2	1	45	60

A second coding has been developed, in order to set an expected operating time, with systemic risks that depend upon the patient’s condition, and can perceptibly affect the operating time, as shown in Table 7.

**Table 7:** Incidence on induction, operating and awakening time for each defined risk

Risk	Incidence on induction time	Incidence on operating time	Incidence on awakening time
Premature	25 %	0	25 %
Baby	25 %	0	0
Heart Disease	100 %	0	50 %
Coagulopathy	50 %	50 %	0
Psycho-Motor Pathology	50 %	0	50 %
Allergies	25 %	0	25 %
Particular Syndromes	25 %	0	25 %
Neuromuscular Pathology	25 %	0	25 %

After defining these incidence parameters, the team applied this model in the specific case of the operating area under analysis. The application to a real scenario allows for modeling finely-grained details, such as the inter-block transportation timings. This specific work, as has been previously stated, results in a complete analysis of the duration of the whole process. Table 8 defines in detail the timing for each task, using the specific case of the selected operating area.

**Table 8:** Execution time for each task defined (minutes)

Task	Timing (min)
Preparation	Start
Transport	From same building = 10
	From a different building = 20
Enter Operating Sector	Std = 10
Preparation Room	Anesthesia Type C = 30
	Anesthesia Type D = 45
Enter Operating Theatre	Std = 10
Anesthesia	An. A = 10
	An. B, C, D = 15
Surgical Operation	EOT (Expected Operating Time decided by the surgeon)
End Anesthesia	Type A = 10
	Type B, C, or D = 15
Exit Operating Theatre	To Intensive Care = 10
	To the Recovery Room = 5
Recovery Room Stay	Std = 30
Transport to Intensive Care	Std = 10
Transport to Ward	To same building = 10
	To a different building = 20

Std = Standard Time

### Optimization model for OR planning

The information gathered from the BPMN design of this process under study, as well as an estimation of the task times, are then integrated into a mathematical optimizational model, aimed at planning the day-to-day planning of OR activities. From a mathematical point of view, the problem can be conceptualized as an optimizational model designed to allocate a given amount of resources (in particular, the operating block times), in the best way possible. (i.e., by maximizing an ‘a priori’ decided objective function).

As already stated, the Operations Research and Management Science Literature is abundant and many papers have been published in the last two decades which attempt to solve this nice combinatorial optimization problem [7, 8].

In general, the OR planning problem can be viewed as consisting of three different and interrelated problems, which also correspond to different levels of decision-making [36, 37]. At a tactical level, the available OR time is divided between the surgical subspecialties that are based on different criteria, such as the total cases per allocated block (i.e., historical utilization), hospital costs, gains per allocated block (i.e., financial criteria), the demand for services (i.e., the waiting list), etc. Once the OR time has been allocated to each surgical group and has been decided upon, the second phase involves the drawing up of MSS, i.e., a cyclic timetable that determines the surgical specialty associated with each OR block or session, during the planning period). When the MSS has been finalized, in the last phase, usually referred to as ‘Surgery Process Scheduling’, the elective cases must be scheduled in each allocated block and the sequence of surgical cases must be determined.

For this study, we have focused our attention on the problem of determining, for a given planning horizon, the assignment of patients to OR blocks, ensuring that the total expected operating time of the patients, scheduled for a specific block, does not exceed the total duration of that OR block (no overtime is allowed in the planning phase).

We assumed a block scheduling operating strategy. This means that for a given planning horizon (let us say, one week), each subspecialty had an ‘a priori’ assigned number of the OR block times for its patients that were scheduled for surgery. An OR block cannot be shared by different subspecialties.

The strategic and tactical decisions pertaining to the availability of ORs during the week, the OR opening hours, and the cyclic timetable, which defines the allocation of the OR blocks to the subspecialties, are known in advance and used in our analysis as input data.

In order to determine the optimal OR planning, different objectives, should be properly considered and evaluated. These objectives may be to maximize the OR utilization rate, to minimize idle time and overtime, to maximize throughput, to minimize patient waiting times, and to analyze and calculate the trade-off between cost, volume, and clinical issues.

In this paper, we have used a 0–1 optimization model partially derived from Tanfani and Testi [38], where the patient-centered objective function aims at minimizing the overall patient cost, which depends on the time taken to meet the clinical needs of the patients on the waiting list. Managing a waiting list, and dealing with both waiting times and prioritization, and then ending with an adequate scheduling of patients, to the precise operating day, is one of the major daily challenges of a modern hospital.

Any attempt at dealing with OR organization and scheduling must be made in parallel with the waiting list management. In the waiting list under study patients are classified into five Urgency Related Groups (URG) and the cost of waiting of each patient is expressed in Need Adjusted Waiting Days (NAWD). The adjusted waiting days are computed by multiplying the urgency coefficient of the URG by the elapsed waiting time of each patient, and by using a prioritization system that has already been validated and is used in the hospital under study [39–41].

Lastly, the optimization model has been designed on a moving target framework and is based on three phases of optimization and re-optimization over the time. Each phase is run in order to schedule different subsets of patients, for different time periods in advance. The time windows to apply the optimization phases are determined in accordance with the urgency class and maximum waiting time of patients waiting as it will be explained in details in the next section.

The study was conducted retrospectively and the Scientific Institutional Board of the Hospital (“Comitato Tecnico Scientifico OPBG”) approved the study, including the use of a set of anonymised data extracted from the OR database, in a retrospective manner (Study Number 201302Q003154). About the clinical care that had generated these data, full information and a family consent always had been obtained. Additionally, none of the results of the research were used for clinical purposes, either during or after the study.

## Results

This model has been applied to devise the OR planning of the hospital under study for a 5-week time period. The results herein reported refers to the planning of the selected operating area that consists of three ORs. The operating theatre is shared by the following 9 surgical subspecialties: Maxillo-Facial & Plastic Surgery, Otolaryngology, General Surgery, Neurosurgery, Urology, Neonatal Surgery, Orthopedics, Gastrointestinal Surgery, and Hepatobiliary Surgery. The available operating rooms are open from Monday to Friday for 12 h daily (divided into morning and afternoon OR block times), besides the assignment by the hospital of the ORs to surgical specialties is given and done on a weekly basis (Table 9).

**Table 9:** Weekly assignment of surgical specialties to ORs and the day

		Monday	Tuesday	Wednesday	Thursday	Friday
OR1						
	8–14 h	Maxillo-Facial Plastic	Otolaryngology	Neurosurgery	Neurosurgery	Maxillo-Facial Plastic
	14–20 h	Neurosurgery	Urology	Neurosurgery	Urology	Maxillo-Facial Plastic
OR2						
	8–14 h	General	Neonatal	Hepatobiliary	Urology	Orthopedy
	14–20 h	General	Neonatal	Hepatobiliary	Urology	Urology
OR3						
	8–14 h	Urology	Gastrointestinal	Orthopedy	Neonatal	Urology
	14–20 h	Urology	Orthopedy	Orthopedy	General	Urology

Data was collected for one year from January 2012 to December 2012. During the period, 3112 elective patients were operated upon. Their distribution into surgical specialties and urgency classes is reported in Tables 10 and 11, respectively. Each surgery was associated to an expected operating time (Table 12). For this model, we used the expected operating time, as decided by the surgeon, which in future, would be the time given to the application of the pathway process, as described above.

**Table 10:** Number of patients operated on for each surgical specialty during the data collection period

Surgical specialty.	Surgeries
Maxillo-Facial Plastic	551
Otolaryngologist	29
General	286
Neurosurgery	89
Urology	1447
Neonatal	82
Orthopedy	106
Gastrointestinal	457
Hepatobiliary	65
Total	3112

**Table 11:** URG class of patients operated on during the data collection period

URG class	Surgeries
A	660
AA	173
B	736
C	583
D	960
Total	3112

**Table 12:** Expected operating time distribution of patients operated on during the data collection period

Expected operating time (in minutes)	Surgeries
0–30	383
30–60	769
60–90	593
90–120	435
120–150	273
>150	659
Total	3112

**Fig. 5:**
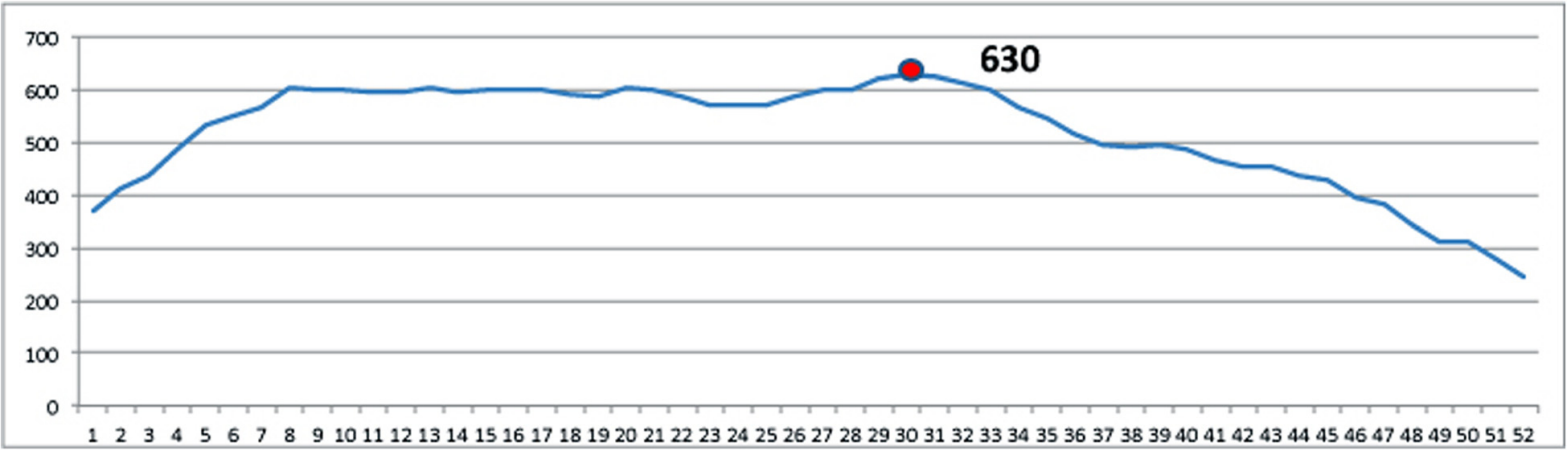
Number of patients on the waiting lists at the beginning of each week

In order to apply and run the model, data concerning the waiting list for a given date is needed. Since the hospital does not yet have an information system, for registering and managing patients on the waiting list for the various subspecialties, we reset the data in order to manually draw a virtual waiting list at the beginning of each week, retrospectively, and consistent with the real data (Fig. 5). A waiting list on 18th June 2012, comprising of 630 patients waiting for surgery, has been chosen, and the OR planning pertains to the week of 23rd July (5 weeks ahead).

The URG characteristics of the patients to be included in the optimization phases, as well as the average utilization of OR blocks for each specialty during each phase, have been set in accordance with the clinicians collaborating in this study and are reported in Table 13.

**Table 13:** Planning phases: patient subsets and average OR occupation

Phase	Period	URG	Avg. utilization
1	5 weeks	B-C-D	50 %
2	3 weeks	A	25 %
3	1 week	AA	25 %

The average utilization is related to the surgery time assigned to each specialty, i.e., to the sum of the total duration of OR blocks ‘a priori’ assigned in the MSS to each specialty. The model gives for each block, a detailed schedule of the set of patients to be operated on, and the resulting OR block occupation.

During the first phase (5 weeks before surgery), 78 patients were scheduled for surgery. Among them, 64, 10, 4 patients belong to URG B, C and D, respectively.

As a result, the occupation time in each block varied since the scheduling depended on the characteristics of the patients that were entered on the waiting list, and of the procedures selected during the first phase. The overall OR time allocated to each surgical specialty was set as a model parameter (to avoid “cannibalism” of the allocated blocks by one specialty over another).

At the ending of the “first phase”, the schedule consisted of the OR blocks that were programmed for 50 % of its capacity, and the waiting list was cleared from the patients selected for the scheduling.

Two weeks later, the second phase started (Table 13). By that day, the waiting list had changed from the day of the first phase, because many new patients, who arrived after the 18th June, were added to the waiting list. Within these new entries, many were attributed non-urgent codes (C or D), but a good number were allocated to a relatively urgent codification (A or B). Of course, the latter patients, especially those with the code “A” had to be considered for being scheduled in the OR with the highest priority. Thus, in the second phase, the model ran, including as a soft constraint, the patients already planned in phase 1, and with the objective of re-optimizing the schedule, while including the patients belonging to URG codes A and B, where appropriate. Of course, the model would extend to codes B and C, and even D patients, if the number of priority patients was low and allowed space in the blocks. The model was run in such manner until a 75 % utilization rate for each specialty was reached. The number of patients that were pre-planned, and then cancelled, or swapped, in the new schedule, should be minimized. In this study, the schedule resulting from phase 2 included 29 additional patients for a total of 107 patients scheduled (3 weeks in advance). Of these, 14, 73, 14, and 6 patients belonged to urgency class A, B, C and D, respectively.

The model gave priority to URG A patients, during any phase it ran. Therefore, and as a result of the first phase planning, there were difficulties in assigning the necessary OR time to some URG A patients, when running the second phase. For that reason, the model allows, in real-time, for the re-allocating of patients of a less urgent code and nature, to another day or week. While running the second phase, and in order to schedule all of the URG A patients, the model postponed 4 patients that were already planned to be in phase 1 to another day or OR; only 1 patient (code C) was cancelled and was moved back to the waiting list.

A comparison of the results reported herein, with the real data of the referenced week, shows that by using the optimization model, allows for the scheduling of about 30 % more patients than in actual practice, as well as to better exploit the OR efficiency, increasing the average operating room utilization rate up to 20 %.

## Discussion

The Bambino Gesù Children’s Hospital is a center of excellence for research and for innovation in pediatric care, and has been the scenario selected for analyzing a surgical reality and describing the OR pathway, and for capturing a set of clinical data that was anonymised and completed for the purpose of running this model. Selecting a children’s hospital was not specific for the study, and it was simply coincidental, in that a large part of the authors have existing working relationships with that hospital.

The standard pathway and the OR optimization model, resulting from this analysis, are not specific to a children’s hospital and can be applied to the reality of other hospitals. There has been, however, one major advantage in using the Bambino Gesù Children’s Hospital as model of OR and surgical reality. In this facility, the ORs are distributed in a few small complexes, rather than in a uniquely large OR area, and therefore, giving the special opportunity of dealing with a limited number of operating rooms, but with a highly complex patient case-mix to start the modeling.

Improving the cost effectiveness of operating room areas in large hospitals, while at the same time, maintaining an excellent quality of care is a modern necessity and a serious challenge. The adoption of standard pathways and processes (clinical pathways) is a necessary evolution to opt for in the future. However, the vast spectrum of procedure types, the unpredictability of the duration of interventions, and taking into account, both last-minute changes and the prioritization of patients on the waiting list, have harmed many attempts at defining a mathematical model in simple theoretical exercises in the past.

In this paper the whole pathway of a patient undergoing intervention (from the time a patient is waiting in ward ready for surgery, to the final step when the intervention is finished, and then when the patient is ready to leave the operating room) has been firstly described, as a standardized sequence including all of the elementary steps or procedures to be taken and associating to each element its own parameters of duration and resources to be used.

In this study BPMN has been adopted as a reference standard to describe the standardized operating pathway since it helps greatly to switch theoretical ideas and research into practical implementation. In this study, the BPMN resource was a great tool used as a communication tool between researchers of different backgrounds (IT, management, or clinical). It was also helpful to identify all of the key variables (staff, resources, actions) that had to be considered in the optimization model. The BPM study enabled a description of each of the processes of each surgical situation. It is, in fact, a generic description, but it can be modeled to every individual patient’s situation. Overall, it is both simple as a path, but complex with a rich combination of variables that make it not only flexible and adaptable for every conceivable intervention type, but to also be able to deliver a duration and a resource use, that is specific to each intervention modeled through that particular sequence. Furthermore, the description of each surgical operation using this approach is made easier. The scheduling of the interventions in the operating theatre makes each “operation item” become an entire description of its specification and its characteristics. Thereby, it facilitates much re-allocating to a given item, to another room, to another time or day, or simply because it contains its own specificities and constraints.

On the other hand, the planning and scheduling of interventions within an operating room area, is not a simple allocation of space and time that is assigned to surgical teams on a given day, but a complex process that starts upstream with the management of a waiting list, where the patients to schedule are queuing until there is an allocated date, and a time and a room in the OR theatre. Last, but not least, the selection of patients on the waiting list is far from a distribution of OR space on a “first come, first served” approach. A set of variables (surgery type, complexity, associated diseases, etc.) and a priority score (clinical relative urgency) is defined for each patient, with many patients arriving last on the waiting list, but having to be served first.

For that purpose, a moving target approach has been applied running the mathematical optimization model, basically based on three phases of optimization and re-optimization over the time. This allows a progressive inclusion of patients onto the schedule, but still leaving space for more urgent patients until the last moment. Thus the re-allocation of patients to another day, or a last-minute reorganization of the OR day schedule, is made possible.

One of the originalities of our approach is that at the time of registration onto the waiting list, both the patient and the intervention variables are entered into the database. This allows one to manage not only the waiting list dynamically, but also reveals an immediately clear idea of the OR scheduling constraints, from the time of insertion on the list. Overall, both processes feed from a single database, with the objective of ending with optimized planning. By allowing a precise OR scheduling, it feeds further up-stream by the managing of the waiting list in an interactive and bi-directional dynamic process.

Last, but not least, by running the model using a set of data extracted from a clinical waiting list database, the OR scheduling showed that the optimization model could end with a proposal for a schedule enrolling of about 30 % more patients than it had been done by a usual clinical allocation. The latter schedule also allowed for a better OR efficiency, increasing the average operating room utilization rate by 20 % and this we suggest should be tested in the reality.

## Conclusions

The present study has resulted in the definition of a specific and detailed pathway for each surgical patient. The tasks, of all of the information necessary for defining the pathway, the human resources involved, the timing and duration of each elementary task, and the specific expected occupation time of the OR, were assessed. The model that was created for this study is original in that, on the one hand, it integrates a standardized pathway that is simple, but it also contains all of the elements of variability. On the other hand, it feeds from a “waiting list database”, with the objective of ending in an optimized allocation of OR resources for registered patients (taking into account their urgency code, in a real-time manner). Lastly, the process between the waiting list and the OR scheduling is interactive, bi-directional, and dynamic, and is a new approach to two major challenges in modern hospitals.

Running the model on real data demonstrated that a 10 % to 20 % increase in operating theatre occupation is possible.

Future work will be devoted to testing the model on a larger set of instances and on additional periods of weeks, using advanced heuristics and metaheuristics methods to deal with the increasing complexity of the instances [42–44]. A sensitivity analysis of the average utilization parameters included in the model is also needed, in order to evaluate the trade-off between the flexibility and the efficiency of the OR plan developed, and to subsequently fine tune these parameters case by case.

As has been previously stated, the complete definition and standardization of such processes is fundamental for healthcare facilities. It is also the first step towards the optimization and dynamic management of operating theatres by means of a continuous software implementation.

Further research and studies are necessary. They must address, for example, the precise execution and implementation of the business process. Furthermore, the same modeling could be used for other operative sectors and then extended, in our case, to the entire pediatric facilities in the hospital.

## Abbreviations

OR: Operating room
BPMN: Business process model & notation
BPD: Business process diagram
MSS: Master surgical schedule
URG: Urgency related groups
NAWD: Need adjusted waiting days
